# Use of Carabids for the Post-Market Environmental Monitoring of Genetically Modified Crops

**DOI:** 10.3390/toxins9040121

**Published:** 2017-03-29

**Authors:** Oxana Skoková Habuštová, Zdeňka Svobodová, Ľudovít Cagáň, František Sehnal

**Affiliations:** 1 Institute of Entomology, Biology Centre CAS, Branišovská 31, 370 05 České Budějovice, Czech Republic; svobodova@entu.cas.cz (Z.S.); frantisek.sehnal@bc.cas.cz (F.S.); 2 Department of Plant Protection, Faculty of Agrobiology and Food Resources, Slovak Agricultural University, Tr. A. Hlinku 2, 949 76 Nitra, Slovakia; ludovit.cagan@gmail.com; 3 Faculty of Science, University of South Bohemia in České Budějovice, Branišovská 31, 370 05 České Budějovice, Czech Republic

**Keywords:** Carabidae, surrogate, post-market environmental monitoring, PMEM, risk assessment, GM maize, functional trait

## Abstract

Post-market environmental monitoring (PMEM) of genetically modified (GM) crops is required by EU legislation and has been a subject of debate for many years; however, no consensus on the methodology to be used has been reached. We explored the suitability of carabid beetles as surrogates for the detection of unintended effects of GM crops in general PMEM surveillance. Our study combines data on carabid communities from five maize field trials in Central Europe. Altogether, 86 species and 58,304 individuals were collected. Modeling based on the gradual elimination of the least abundant species, or of the fewest categories of functional traits, showed that a trait-based analysis of the most common species may be suitable for PMEM. Species represented by fewer than 230 individuals (all localities combined) should be excluded and species with an abundance higher than 600 should be preserved for statistical analyses. Sixteen species, representing 15 categories of functional traits fulfill these criteria, are typical dominant inhabitants of agroecocoenoses in Central Europe, are easy to determine, and their functional classification is well known. The effect of sampling year is negligible when at least four samples are collected during maize development beginning from 1 April. The recommended methodology fulfills PMEM requirements, including applicability to large-scale use. However, suggested thresholds of carabid comparability should be verified before definitive conclusions are drawn.

## 1. Introduction

Although genetically modified (GM) crops are generally considered safe for non-target arthropods [1], there are still uncertainties regarding the long-term effects caused by the accumulation of miniscule changes in the agroecosystem. Science-based post-market environmental monitoring (PMEM) is therefore required for GM crops by EU legislation (Directive 2001/18/EC, [2,3]). PMEM aims to identify risks that did not become evident during the pre-market risk assessment. Consequently, PMEM results are expected to provide a basis for subsequent regulatory decisions, including the prolongation and modification of the monitoring plans. The detection of adverse changes in the environment may trigger additional research that could eventually lead to the withdrawal of approval for GM crops [4].

There has been much discussion about the PMEM of GM crops, but a general PMEM plan accepted by regulators, scientists, and the agricultural biotech industry is still lacking [5]. PMEMs are a legal requirement, and consist of two conceptually different components: (a) case-specific monitoring; and (b) general surveillance (GS) [6], which is the subject of this paper. GS has an unspecified nature. It is part of an inherent challenge for PMEM, because the currently applied GS methodology may not be sensitive enough [7]. The collection of empirical data must be improved and proper baselines of GM-independent insect fluctuations must be established. Since it is impossible to monitor all components of the ecosystems, the selection of surrogate species representing valid entities of the environment is of primary importance.

Five reports [8,9,10,11,12] have proposed that generalist natural enemies are suitable surrogates for the GS component of PMEM. The carabids (Coleoptera: Carabidae) are particularly appropriate because they are species rich, abundant, and functionally diversified in arable habitats all over world [13]. More than 600 species have been recorded in Central Europe [14] and keys for species identification are available. With respect to the method of arthropod collection, pitfall trapping of carabids has a higher capacity to detect differences than the visual monitoring used for the plant-dwelling arthropods [8,9]. Carabids have been considered as bioindicators of the environmental impact of agricultural practices [13,15,16,17,18,19], including the cultivation of GM crops [9,20,21]. Carabids living in fields planted with GM crops are directly and/or indirectly exposed to the products of transgenes [20], depending on their feeding behavior, which ranges from obligate phytophagy to obligate zoophagy, with most granivorous species belonging somewhere in the middle of this continuum [22,23,24].

Carabids play an important role in agroecosystems by contributing to the elimination of a wide variety of weed seeds [25,26,27] and pest insects [28]. They tend to have one generation per year. Some reproduce during spring and complete their development in winter; others breed in autumn and hibernate mainly as larvae. Adults of many species reproduce in spring, aestivate in summer, and reproduce again in the autumn [13]. The body size, habitat, and humidity affinities affect life history parameters and ecological interactions of every species [29]. Functional classification of carabids facilitates the assessment of their roles and their occurrence in agroecosystems [30].

Our study evaluated carabids with respect to body size, habitat and humidity affinities, breeding period, and food specialization. This approach requires identification and counting of captured species, and then choice of indicator species that represent crucial functional traits and are sufficiently widespread for statistical analyses [18]. Profound changes in the representation of functional traits alter the role of carabids in the ecosystem and may have considerable environmental consequences. Simple counts of captured beetles do not disclose these environmental impacts because species with different or unknown traits are mixed up [17].

The importance of species-based analysis is supported by the database of the non-target arthropods species proposed for the environmental risk assessment of GM crops in the EU [31]. Several authors have recommended functional analysis for the comparison of insect communities [32,33,34]. In our analysis of both quantitative and qualitative changes of the carabid community, we combined data on species abundance with information on their functional traits. In this paper, we demonstrate that combining the population size assessment with analyses of functional traits generates a robust and testable method for the comparison of carabid communities.

Since the only GM crop approved for commercial cultivation in the EU is the lepidopteran-resistant maize MON 810, which is grown in five European countries including the Czech Republic and Slovakia [35], we concentrated on carabid communities in maize fields to study the size of the data (how many species, individuals) to be used in GS protocols in the framework of PMEM. Several kinds of maize cultivars, including three GM cultivars, were grown in fields 2–200 km apart. Species diversity and abundance were examined with respect to environmental variables (locality, year, and sampling date) and analyzed in relation to the species and trait categories (body size, habitat and humidity affinities, breeding period, and food specialization) in order to identify optimal conditions for the comparison of communities from different fields and years.

## 2. Results

### 2.1. Characterization and Quantitative Comparison of Carabid Communities

The sum of catches in all localities totaled 58,304 individuals belonging to 86 species. Within functional traits, the abundance of one category usually prevailed, but the species richness was highest in different categories. This applies to number of individuals and species richness in the size categories B and C, species preferring open biotopes and species with low habitat preferences (eurytopic), and hygrophilous and eurytopic species (humidity affinity). The abundances of spring and autumn breeders were very similar, while the numbers of species differed substantially. A similar situation was found for the carnivorous and omnivorous species (Table 1).

The Simpson dominance and Berger–Parker indices were highest in locality SB3, where 80% of individuals were identified as *Pterostichus melanarius*. The second highest index was found in WS with a dominance of *Pseudoophonus rufipes* (70%), and the third highest in SB2, where *Poecilus cupreus* represented 60% of individuals. The dominance of *P. rufipes* in CB (47%) and of *P. melanarius* in SB1 (32%) was less pronounced. This was reflected in the species evenness, which was highest in SB1 and lowest in SB3 in which species evenness was very similar to WS. The Margalef index detected highest ratio between the number of species and the abundance in SB2 (59 species, 22,015 individuals), followed by SB1 (35 species, 5484 individuals), CB (35 species, 5831 individuals), WS (34 species, 9401 individuals), and SB3 (34 species, 15,573 individuals, Table 2).

The Jaccard and Sørensen–Dice indices showed dissimilarity between communities when all localities were compared. The most similar communities were found in the three geographically closest localities in South Bohemia (Table 3).

### 2.2. The Effect of Locality, Year, and Sampling Date on Carabid Communities (All Data Included)

The location explained 16.3% and 23.8% variability in the distribution of carabid species and functional categories, respectively. In the species-based canonical correspondence analysis (CCA), the WS locality explained 10.2% (*F* = 169.0, *p* = 0.001), CB 8.8% (*F* = 144.4, *p* = 0.001), SB3 3.3% (*F* = 50.6, *p* = 0.001), SB2 1.9% (*F* = 29.1, *p* = 0.001), and SB1 1.9% (*F* = 29.0, *p* = 0.001) variability. The trait-based CCA yielded higher values: SB3 14.9% (*F* = 259.8, *p* = 0.001), WS 8.9% (*F* = 148.0, *p* = 0.001), CB 4.9% (*F* = 75.3, *p* = 0.001), SB2 4.6% (*F* = 71.5, *p* = 0.001), and SB1 2.3% (*F* = 33.1, *p* = 0.001).

The year explained 1.0% (*F* = 14.9, *p* = 0.001) and 1.9% (*F* = 27.8, *p* = 0.001) of variability in the species- and trait-based CCA, respectively. In the species-based CCA, each of the time series S and A explained 3.6% of variability (S: *F* = 56.3, *p* = 0.001, A: *F* = 54.7, *p* = 0.001). The joint analysis of both series proved their close correlation. In the trait-based CCA, the time series S explained 4.3% (*F* = 67.1, *p* = 0.001), and the time series A explained 4.7% of variability (*F* = 70.4, *p* = 0.001). The joint analysis of both series showed they were correlated, sharing 4.1% of the variability they explained. Only time series A was used in subsequent modeling.

### 2.3. Three Possible Ways of Using Carabids in PMEM

**Figure 1:**
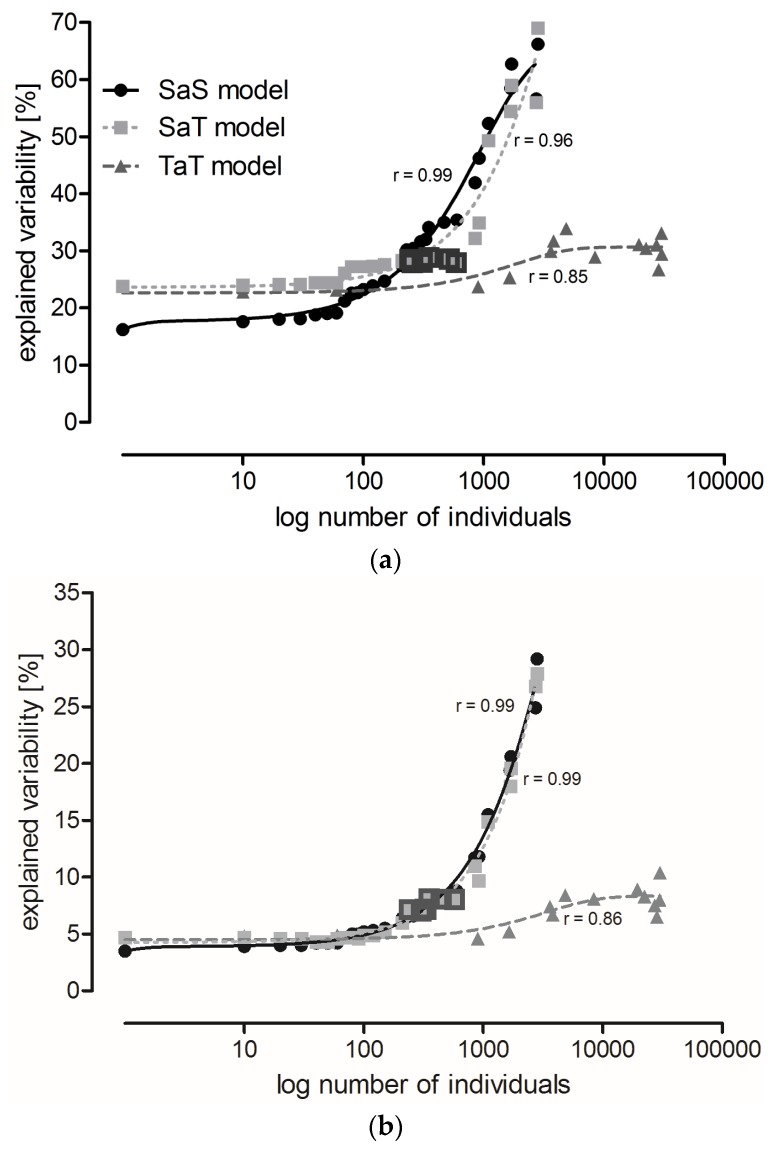

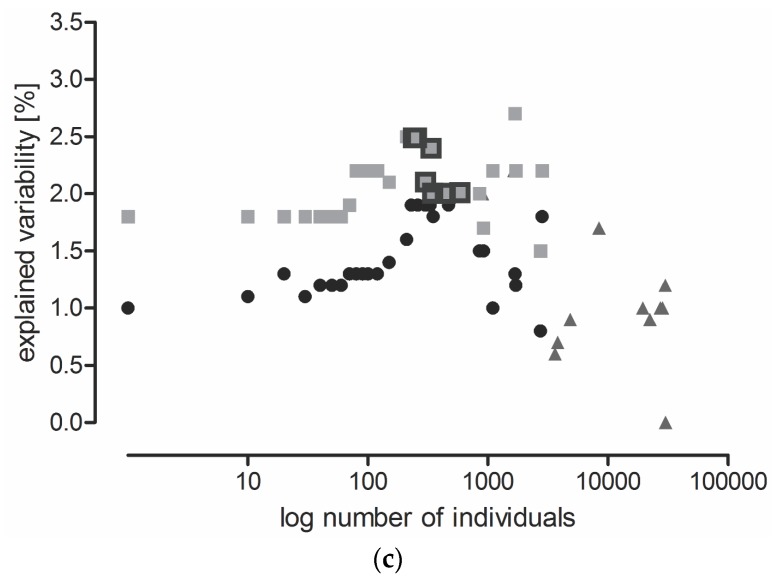
Variability in the carabid grouping composition explained by: (**a**) locality; (**b**) sampling date (time series A); and (**c**) year in the SaS model (solid lines), SaT model (dotted lines), and TaT model (broken lines). Each point represents a Canonical Correspondence Analysis (CCA). Consecutive points were calculated allowing the same data to be analyzed, but the least abundant species (SaS and SaT models) or categories of functional traits (TaT model) were eliminated, and CCA was performed at the species level (SaS model) or at the level of functional traits (SaT and TaT models). Individuals were gradually eliminated with a cut-off level of 10. In the SaS and SaT models, species elimination proceeded until the three most common species were left. In the TaT model, the categories of functional traits were eliminated until the three most common categories remained. Highlighted points represent analyses where species with abundance from 230 to 600 individuals are preserved (explained in Section 2.3 Three Possible Ways of Using Carabids in PMEM).

Variability explained by localities remained relatively stable in the SaS and SaT models (see Section 5.3) until species with abundance lower than 150 and 600 individuals, respectively, were disregarded (Figure 1a). Subsequent step-wise elimination of the more abundant species resulted in a steep increase in explained variability (the difference between variability explained by two adjacent points in the graphs significantly increased, SaS: *F*_1,25_ = 11.69, *p* = 0.002, SaT: *F*_1,25_ = 35.84, *p* < 10^−5^). Only a small increase in explained variability was observed with the TaT model (Figure 1a). The curve derived from the SaS model intersects the curves of the TaT and SaT models at the points corresponding to species represented by 117 and 229 individuals, respectively, where the variability explained by the SaS model exceeded the variability explained by both the SaT and TaT models.

Variability explained by localities was about four-times higher than that explained by the sampling date (time series A) in the analysis that included all species. However, variability explained by the sampling date increased faster with species elimination, and eventually became half that explained by the localities (Figure 1b). The increase in variability explained by time series A was similar in the SaS and SaT models. The smallest increase in variability was observed in the TaT model (Figure 1b). Variability explained by years was very low in all models (Figure 1c).

The lowest percentage of variability explained by localities occurred in the TaT model and in the SaS model at the beginning of modeling (*x* ≤ 150), suggesting that these two approaches were most appropriate to compare the least locality-dependent environmental impacts. However, these procedures required the determination of all individuals to the species level, and in the case of the TaT model also their classification into categories of functional traits. Neither of these requirements can be fulfilled in routine practice. However, variability explained by localities in the SaT model was at *x* = 229, exceeded by variability explained by the SaS model, and then increased slowly up to *x* = 600 (Table 4). Species with abundance lower than 230 (in total for all localities) could be excluded from the analysis, while species with abundance higher than 600 (10 species, 14 categories) had to be included (this range is highlighted in Figure 1) to avoid a high increase of variability. Six species were found in all localities and their total abundance surpassed 600 (Table 5). The SaT model showed conditions that have to be fulfilled for reliable comparison of different localities in routine practice.

**Figure 2:**
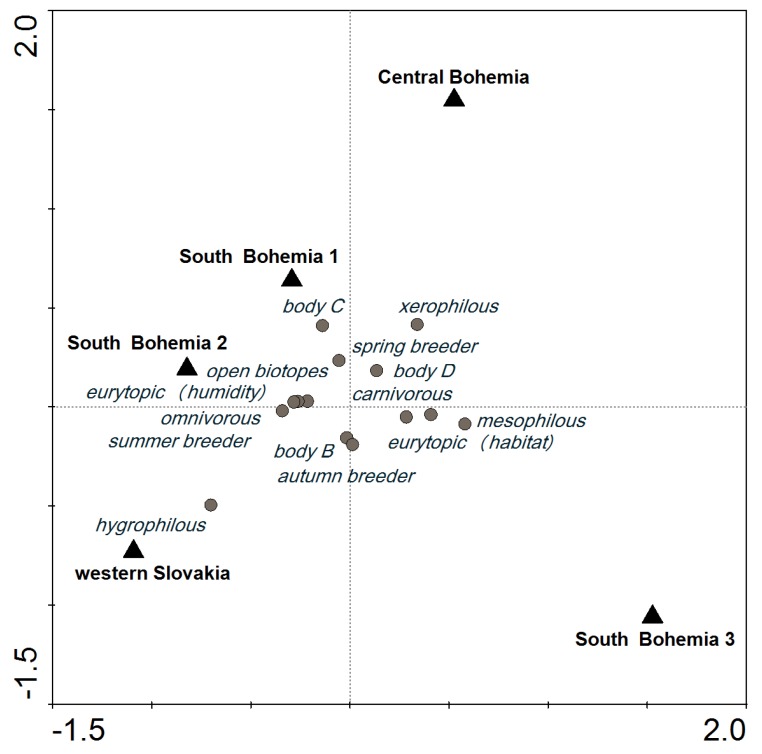
CCA ordination diagram showing the importance of locality for functional trait categories of carabids that reached an abundance of at least 600 (10 species, 14 categories) based on the SaT model (explained in Section 5.3 Data Analysis).

In the SaT model at a cut-off level of 600, most of the categories of functional traits were distributed around the center of the ordination diagram; their presence was similar in all localities. However, the incidence of hygrophilous species tended to be higher in WS, and species in body size category C were most common in SB1 (Figure 2). Similar carabid groupings were found in SB1 and SB2, which were about 2-km apart, including 1 km of a forest. A similar species composition was also found in SB3 (Table 3), but the species abundance was different (Figure 2).

**Figure 3:**
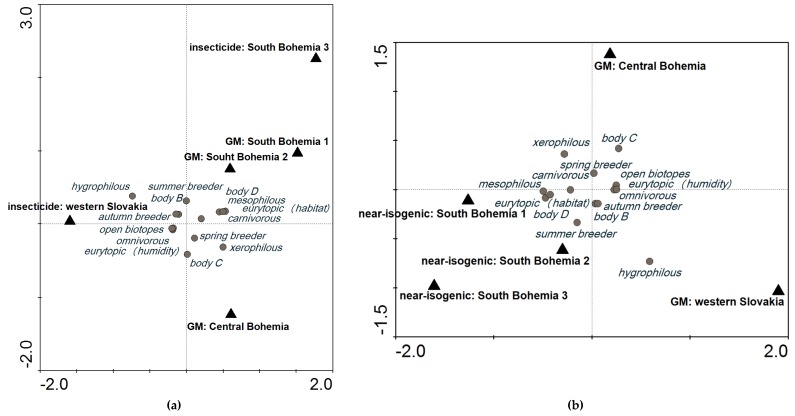
Comparison of plots with different treatments: (**a**) plots with GM events (SB1, SB2, and CB) compared with plots treated with insecticide (SB3 and WS) at a cut-off level of 600; and (**b**) plots with GM events (CB and WS) compared with plots with near-isogenic cultivars (SB1, SB2, and SB3) at a cut-off level of 600. CCA ordination diagrams are based on the SaT model (explained in Section 5.3 Data Analysis).

When we compared plots with GM events and plots treated with insecticides at a cut-off level of 600, the variability explained by these localities was 20.7%. It was lower than baseline (Figure 1a: SaT model, *x* (no. of individuals) = 600, *y* (explained variability) = 28.1%), indicating low probability of an impact of GM maize on the carabid groupings in these localities (Figure 3a). When GM events were compared with near-isogenic cultivars, variability among these plots in different localities was 28.9% (Figure 3b), which is still around the level of variability explained by different localities. A higher difference would indicate that the GM crop had an impact on the agroecosystem.

## 3. Discussion

Development of a PMEM method that is applicable in a large geographic area at reasonable cost is very challenging. Such a method should be based on indicators that are relatively easy to monitor, occur in vast territories from spring to autumn, and are exposed to the products of transgenes in GM crops. Carabids fulfill these requirements, but the feasibility of their monitoring has not been sufficiently analyzed. Therefore, our study focused on the nature and size of data needed for reliable distinctions between carabid communities in different localities, years, and sampling dates. Carabid assemblages were analyzed in respect to the species composition and functional traits. This approach was preferred over the analysis of total abundance of the carabid family.

### 3.1. Carabid Communities in Maize Fields

To facilitate comparison with other studies, we used several indices to characterize carabid communities in different localities. The values of diversity indices were more or less in the range of those reported across Europe [17,32,36,37]. Similarity indices based on the qualitative species comparison declined with the distance between compared localities. Conversely, functional diversity was similar in remote localities; all important categories of functional traits were present in all localities (TaT model, Figure 1a).

The Berger–Parker index is an effective, simple tool for monitoring impaired biodiversity in soil ecosystems due to human disturbances [38]. Index values increase from undisturbed to disturbed areas. In the present study, values ranging from 0.32 (SB1) to 0.70 (WS) were typical for sites with agricultural management and indicate the prevalence of one species. Use of this index can facilitate interpretation of soil biodiversity patterns in the context of ecosystem management and conservation. The index of species evenness was low. It ranged from 0.38 (SB) to 0.59 (SB1), and reflected dominance by one species and very low abundance of others [39]. 

The dominance of a few species varied in their dependence on locality, and annual changes in environmental and anthropogenic factors [36,37,39]. The 16 most abundant species in our study are common in the fields of Central Europe [17,34,36,37,39,40], and some are also found in southern Europe [9,11,41], the United Kingdom [32], and Balkan [42]. This suggests similarity in agrocoenoses across a large area; usually 10–20 frequently occurring species rotate in the position of the most abundant species. However, only five of these species (*C. fuscipes*, *H. affinis*, *P. cupreus*, *P. melanarius,* and *T. quadristriatus*) were among the 10 most common species in all localities. According to literature sources [9,11,17,32,34,36,37,39,40,41,42] and our experience, we suggest that it is appropriate to compare the abundance of a group of 10–20 common species based on their functional classification. A similar conclusion has been reached by other authors [11].

### 3.2. Variability Explained by Locality and Environmental Variables

Site location is by far the most important source of variability [8]. Locality is therefore important for making a baseline between background variability caused by other factors than the studied treatment. Several studies have compared trials conducted under different management regimes and used different statistical methods to define the sample size sufficient to detect impacts of GM crops on the abundance of arthropods [8,9,11,43,44,45]. We used CCA with three types of gradual species reduction or categories of functional traits to identify the minimum sample size that would sufficiently minimize the locality-dependent variability. Modeling was based on species abundance, and representation of categories of functional traits (explained in Section 5.3 Data Analysis). When all data were included in the analysis (first point in Figure 1a), the percentage of variability explained by localities was lowest in the SaS model, which showed the importance of species of low abundance for the similarity assessments of distant localities. The SaT model showed that variability explained by localities remains relatively low when species with abundance lower than 230 individuals are neglected. Species can be further eliminated from the analysis up to a threshold of 600 individuals per species. Exclusion of species with higher abundance causes a very steep increase in variability among localities. We do not recommend analyses based on less than 10 most common species. A trait-based analysis with the most common species is a compromise that can be utilized for large scale PMEM (limitations of PMEM are discussed in [10]). Species with an abundance lower than 230 can be excluded from the analysis, while those with an abundance higher than 600 must be preserved to avoid a high increase of unwanted variability.

We observed baseline background variability for carabid grouping comparisons in five different localities. If the percentages of variability explained by the GM and non-GM treatments were higher than the baseline for these localities, an impact of the GM crop on the agroecosystem may be indicated. The baseline for similar localities is lower than for the less similar localities. Thus, it is necessary to determine the approximate baseline variability for the examined localities.

Maize phenology was not crucial for the carabid grouping (shared variability between time series S and A). We propose to follow a time series based on the calendar date rather than on the date of maize sowing, as this varies between localities and years. The day 1 April seems to be a reliable landmark for Central Europe and probably for most of Europe.

Differences of 14 days between corresponding samplings in different localities were not uncommon in our study. Given the low percentage of variability explained by the sampling date, the precise timing of samplings in different localities is not needed. Our conclusions are mostly based on four samplings per season. Since the abundance and species composition fluctuate during the season [36], a minimum of four samplings per season are required. A reduced sampling number may lead to a considerable loss in the capacity to detect differences [45]. Most studies have confirmed the highest carabid abundance around the time of maize flowering [11,46], although this is not the rule [10]. It is advisable to cover the first part of the season (until grain development, [21]).

Unlike the findings of previous studies [8,11], the effect of different sampling years was relatively low and can be neglected. It seems that direct comparison with a current non-GM crop baseline should be used if available, but reference can also be made to historical baseline data [7].

### 3.3. The Applicability of Our Findings for GS in PMEM of GM Maize

Recommendations based on the findings of our study should be taken into account when designing statistical comparisons in PMEM. If any unintended effect is observed, the recommended data analysis will help to determine whether the adverse effect is associated with the use of a GM crop or whether it is a consequence of other environmental factors [47].

Many authors have highlighted the importance of field size and the availability of non-crop habitats adjacent to the field [36,39]. Those factors should be taken into account when differences among localities are to be interpreted. Multivariate analysis, as used in our study, is a multidimensional tool that considers the effects of many variables, and is appropriate for evaluating and subtracting the effects of these covariables.

Although the European Food Safety Authority (EFSA) claims that GS is not necessarily crop or event specific [4], we are convinced that GS methodology could be the same for different crops, but the direct comparison of carabid assemblage in various crops species, and the effects of agricultural practice are scientifically not defensible because the species and abundance composition is largely affected by crop type even in one locality. This was clearly shown for GM and near-isogenic cultivars of maize, beet, and oil seed rape [32].

## 4. Conclusions

The conservation of natural enemies, which as an important component of biological control, can be accepted as the endpoint of PMEM. We propose that these are monitored by analyzing captured carabid species with respect to their abundance and ecological functions. Reliable results are obtained with commonly occurring and abundant species representing important functional categories. Field location is the main factor limiting the detection of changes caused in carabid assemblages by other factors, including the introduction of GM crops. The location effect is preserved at a tolerable level by including only species that occur in relatively high numbers in all examined sites. We demonstrate that, in the case of our model, the inclusion of 10 species, each represented by ≥600 individuals (total count from all sites) is essential, while 70 species with less than 230 individuals each could be excluded without a significant increase in variability observed. The geographically closest localities with similar environmental properties should be preferably compared to reduce differences. At least four samplings during the season are recommended on similar, but not necessarily the same, dates. A time series based on the calendar date can be followed, and data from different years can be combined. Data from independent carabid analyses in maize fields (and possibly in the plantations of some other crops) can be included in future analyses and further increase the precision of PMEM. The proposed method is a compromise that enables the detection of small but meaningful differences at maximally reduced labor costs.

## 5. Materials and Methods

### 5.1. Experimental Localities

Field trials were performed in localities designated as South Bohemia 1, 2, and 3 (SB1, SB2, and SB3); Central Bohemia (CB); and western Slovakia (WS). Basic features of all localities are summarized in Table 6; details on WS site are provided here. Carabid data obtained from the sites SB1, SB2, and CB have been published (see references in Table 6) and are not evaluated from the perspective of the present study.

The WS trial was performed in a field previously planted with winter wheat. In the first trial, three treatments were applied in four replicates (12 plots in total, 30 × 30 m each). In the second trial, two treatments were tested in 10 replicates (20 plots in total, 10 × 10 m each). Each plot was isolated by a 1- and 5-m wide strip of barley in the first and second trial, respectively. Fertilization with urea (CH_4_N_2_O, 100 kg/ha) and Polidap (18% N, 46% P_2_O_5_, 200 kg/ha) was applied before sowing in 2014, and with urea and NPK 15-15-15 (N [NH4^+^, NO3^−^], P_2_O_5_, K_2_O, 150 kg/ha) in 2015. Trials were treated with the pre-emergent selective herbicide Dual Gold (s-metolachlor, 1.25 L/ha) and Mustang (florasulam, 0.8 L/ha) on 7–26 May 2014, and with Wing (dimethenamid-p, pendimethalin, 4.0 L/ha) on 7 May 2015. All neighboring fields in both years were sown with oilseed rape. Carabid assemblages from the two trials were combined.

### 5.2. Capture and Identification of Carabids

Pitfall traps (9-cm diameter, 0.5–1 volume) were supplied with about 300 mL 10% NaCl and 2–3 drops of detergent (SB1, SB2, SB3, CB), or with ethylene glycol and water 1:1, (WS), covered with aluminum coping and exposed for 7 days. Different numbers of pitfall traps were used (calculated per ha: seven traps in SB1, four in SB2, eight in SB3, 25 in CB, and 19 in WS; 3669 pitfall trap collections in total).

Samples in SB1, SB2, SB3, and CB were collected at maize stages BBCH 09, BBCH 16, BBCH 65, and BBCH 87 [51]. In WS, samples were collected every other week at maize stages BBCH 09, BBCH 11, BBCH 13, BBCH 17, BBCH 34, BBCH 53, BBCH 63, BBCH 69, BBCH 79, and BBCH 89 (sampling dates are provided in Table S1, Supplementary Materials).

Carabids were stored in 70% ethanol and identified to species level [52] (Table S2, Supplementary Materials). Body size, humidity, habitat affinities, breeding period [52], incidence [53,54], and food specialization [23] were determined for each species (Table S3, Supplementary Materials).

### 5.3. Data Analysis

We used the following ecological indices to compare the diversity of carabid communities in different localities: Berger–Parker index, Margalef index, Simpson dominance index, and Species evenness. The Jaccard index and Sørensen–Dice index were applied to assess the similarity of communities in different localities [55].

Carabid distribution was analyzed using multivariate analysis (Canoco software for Windows 4.5, Plant Research International, [56]). The analysis concerned the abundance of species and their placing in the functional trait categories for body size, habitat and humidity affinities, breeding period, and food specialization. The detected gradient length (4.9) in the detrended correspondence analysis (DCA: 0.001 attributed to each value, detrending by segments, log transformation: *x*’ = log (*x* + 1), downweighting of rare species) of distribution trends and the characterization of data enabled us to use canonical correspondence analysis (CCA: 0.001 attributed to each value, log transformation, Hill’s scaling). The effects of geographic localization of each locality (dummy variables), year (dummy variables), and sampling date were tested (environmental variables). A two-time series was used to test the effect of sampling date: (1) time series, S: number of days from the sowing day, marked as number 1; and (2) time series A: number of days from 1 April, which was classed as Day 1. The day 1 April was selected based on the agro-technical term of maize sowing. The earliest possible term for sowing in Central Europe is around 5 April [57]. The joint explanatory effect of these two variables was assessed by the analysis of variability explained with the time series S and A in two separate CCAs, and together in a single CCA (variance partitioning procedure, [56]). The significance of the effects of environmental variables was tested by subtracting the effect of covariables (CCA in partial shape, Monte Carlo permutation tests, MCPT: 999 permutations, unrestricted permutations, forward selection). Covariables are environmental variables whose influence is subtracted before that of variables of interest is investigated [56]. Covariables were those environmental variables mentioned above whose effect were not tested in certain CCAs.

Variability explained by environmental variables in CCA was compared for three different types of gradual elimination of individuals with a cut-off level of 10:

1. SaS model: the least abundant species were eliminated and a CCA was performed at the species level (Table S4);
2. SaT model: the least abundant species were eliminated and a CCA was performed at the level of functional traits (Table S5); and
3. TaT model: the least frequent categories of functional traits were eliminated and a CCA was performed at the level of functional traits (Table S6).

In the SaS and SaT models, species elimination proceeded until three most common species remained. In the TaT model, the categories of functional traits were eliminated until the three most common categories remained. Variability explained by models was compared using the curve (two-phase exponential association, coefficient of correlation r, Graph Pad Prism 4.5, [58]). One-way ANOVA (F-tests accompanied by degrees of freedom and degrees of freedom of the error) was applied to compare differences in variability explained during modeling [59].

We defined baseline as a background variability that is caused by other factors (covariables in multivariate analysis) than the variables (GM vs. non-GM) whose effect is important for the purpose of the study. We hypothesize that when we know baseline, it is possible to distinguish between the background variability and variability caused by growing GM maize. The example of separation of effect of baseline from effect of GM maize is presented in Figure 3a,b. The carabid grouping in GM maize is compared there with carabid groupings in plots treated with insecticides (as they are applied in most of maize cropping systems) and plots with near-isogenic cultivar, respectively.

## Figures and Tables

**Table 1:** Quantitative composition of carabids for functional traits in the localities South Bohemia 1 (SB1), 2 (SB2), and 3 (SB3); Central Bohemia (CB); and western Slovakia (WS).

Trait	Category ^1^	SB1		SB2		SB3		CB		WS		Total	
		Individuals	Species	Individuals	Species	Individuals	Species	Individuals	Species	Individuals	Species	Individuals (%)	Species (%)
Body size													
A		36	1	14	3	2	1	0	0	3	2	55 (0.1)	4 (5)
B		3692	8	18,349	10	14,728	10	2274	7	7214	13	46,257 (79)	19 (22)
C		456	17	2660	34	409	15	3101	18	1765	12	8391 (14)	44 (51)
D		1300	9	992	12	434	8	456	10	419	7	3601 (6)	19 (22)
Habitat affinity													
Silvicolous		59	6	635	15	130	7	66	3	4	7	894 (2)	19 (22)
Open biotopes		2673	16	4861	30	1437	17	4302	22	9009	23	22,282 (38)	44 (51)
Eurytopic		2752	13	16,519	14	14,006	10	1463	10	388	7	35,128 (60)	23 (27)
Humidity affinity													
Hygrophilous		329	15	2563	24	404	16	133	11	1417	8	4846 (8)	34 (40)
Mesophilous		2038	6	3163	11	12,855	6	1161	4	275	6	19,492 (33)	15 (17)
Eurytopic		2288	10	15,622	14	1834	9	3489	10	6935	8	30,168 (52)	17 (20)
Xerophilous		829	4	667	10	480	3	1048	10	774	12	3798 (7)	20 (23)
Breeding period													
Spring		2340	23	17,356	42	2080	24	3490	25	1997	19	27,263 (47)	61 (71)
Summer		141	3	512	6	129	3	241	6	609	4	1632 (3)	8 (9)
Autumn		3242	14	5146	21	13,610	11	2434	12	7232	16	31,664 (54)	29 (34)
Food specialization													
Carnivorous		4258	24	7068	35	13,829	24	2322	20	2418	21	29,895 (51)	55 (64)
Omnivorous		1226	11	14,945	23	1743	9	3508	14	6983	13	28,405 (49)	29 (34)
Granivorous		0	0	2	1	1	1	1	1	0	0	4 (0.007)	2 (2)

^1^ Body size (mid-range): A: ˃22 mm, B: 11–21.9 mm, C: 6–10.9 mm, D: <5.9 mm; Silvicolous: preferring woodlands; Open biotopes: preferring open areas; Eurytopic: adaptable to various environmental conditions; Hygrophilous: preferring moist places; Mesophilous: preferring intermediate or moderate environmental conditions, avoiding extremes of moisture or dryness; Xerophilous: preferring dry environmental conditions.

**Table 2:** Mean (±SE) indices of carabid diversity in the examined localities per year.

Locality	No. of Tested Years	Simpson Dominance Index (D)	Berger–Parker Index (D)	Species Evenness (E)	Margalef Index (DMg)
SB1	1	0.18	0.32	0.59	3.95
SB2	3	0.37 ± 0.08	0.60	0.44 ± 0.07	4.25 ± 0.55
SB3	3	0.53 ± 0.15	0.80	0.38 ± 0.13	2.60 ± 0.23
CB	2	0.28 ± 0.01	0.47	0.53 ± 0.03	3.38 ± 0.21
WS	2	0.48 ± 0.08	0.70	0.39 ± 0.07	3.05 ± 0.30

**Table 3:** Similarity matrices of Jaccard and Sorensen–Dice indices between carabid communities in the examined localities. Highest values for both indices are in bold.

Jaccard Index (JS)						Sørensen–Dice Index (DS)					
Locality						Locality					
	SB1	SB2	SB3	CB	WS		SB1	SB2	SB3	CB	WS
SB1						SB1					
SB2	**0.34**					SB2	**0.51**				
SB3	0.32	0.32				SB3	0.48	0.49			
CB	0.26	0.24	0.23			CB	0.41	0.39	0.38		
WS	0.17	0.17	0.21	0.28		WS	0.30	0.29	0.34	0.43	

**Table 4:** Changes in the variability explained by environmental variables in the SaS, SaT, and TaT models (see Section 5.3 Data Analysis) in CCA between cut-off levels 230 and 600 individuals per species. Values based on data are given before parentheses and values in parentheses are based on values interpolations from the constructed curves.

Environmental Variable	SaS	SaT	TaT
Locality	5.2 (12.1)	0.1 (6.5)	n.a. ^1^ (1.2)
Time series A (Sampling date)	2.3 (3.6)	0.9 (3.1)	n.a. (0.3)
Year	0.1 (n. a.)	0.5 (0)	n.a. (0.1)

^1^ n.a.: not available.

**Table 5:** Species with abundance higher than 230 included in the CCA analysis of the SaT model (explained in Section 5.3 Data Analysis) and their functional classification (explained in footnote of Table 1). Species with abundance higher than 600 are highlighted in bold. Underlined species were sampled in all localities.

Species	Total Abundance	Body Size	Habitat Affinity	Humidity Affinity	Breeding Period	Food Specialization
*Agonum muelleri*	256	C	Eurytopic	Hygrophilous	Spring	Carnivorous
***Anchomenus dorsalis***	1099	C	Open biotopes	Hygrophilous	Spring	Carnivorous
*Bembidion lampros*	462	D	Open biotopes	Eurytopic	Spring	Carnivorous
***Bembidion quadrimaculatum***	1680	D	Open biotopes	Eurytopic	Spring	Carnivorous
*Brachinus crepitans*	348	C	Open biotopes	Xerophilous	Summer	Carnivorous
*Brachinus explondes*	294	D	Open biotopes	Hygrophilous	Spring	Carnivorous
***Calathus fuscipes***	2811	B	Open biotopes	Xerophilous	Autumn	Carnivorous
*Carabus granulatus*	596	B	Silvicolous	Hygrophilous	Spring	Carnivorous
*Clivina fossor*	325	C	Open biotopes	Hygrophilous	Spring	Carnivorous
***Harpalus affinis***	920	C	Open biotopes	Eurytopic	Spring/summer/autumn	Omnivorous
***Harpalus rubripes***	2734	C	Open biotopes	Eurytopic	Spring	Omnivorous
***Poecilus cupreus***	15,975	B	Eurytopic	Eurytopic	Spring	Omnivorous
***Poecilus versicolor***	1710	C	Open biotopes	Hygrophilous	Spring	Carnivorous
***Pseudoophonus rufipes***	7871	B	Open biotopes	Eurytopic	Autumn	Omnivorous
***Pterostichus melanarius***	18,297	B	Eurytopic	Mesophilous	Autumn	Carnivorous
***Trechus quadristriatus***	841	D	Open biotopes	Mesophilous	Autumn	Carnivorous

**Table 6:** Basic features of the examined localities and information on field trials in the localities South Bohemia 1 (SB1), 2 (SB2), and 3 (SB3); Central Bohemia (CB); and western Slovakia (WS).

Features	SB1	SB2	SB3	CB	WS
Timing (sowing–harvest, maize stage during harvest)	2002 (15.5–17.9. (BBCH 87))	2003–2005	2009–2011	2013–2014	2014–2015 (2014: 28.4–29.10. 2015: 5.5–30.10. (2nd trial), 4.11. (1st trial) (BBCH 89))
GPS coordinates	48°97′ N 14°44′ E	48°58′ N 14°24′ E	48°59′ N 14°20′ E	50°09′ N 15°11′ E	48°34′ N 17°43′ E
Altitude (m a.s.l.)	381	409	420	285	160
Climatic region	Moderately warm humid	Moderately warm humid	Moderately warm humid	Warm, slightly dry	Warm, moderate arid
Average annual temperature (°C)	8.1	8.1	8.1	8.9	9.2
Average annual precipitation (mm)	623	623	623	596	593
Prevalent soil type	Cambisol, sandy loam brown	Cambisol, sandy loam brown	Medium-weight, mildly humid clay-loam brown	Medium-grained black floodplain from debris	Loamy luvic chernozem
Trial area (ha)	7.6	14	15	4.38	2.9 (1st trial); 0.52 (2nd trial)
No. of plots (plot size in ha)	10 (0.5)	10 (0.5)	25 (0.5)	54 (0.054)	12 (0.09, 1st trial); 20 (0.01, 2nd trial);
No. of pitfall traps per plot/total amount	5/50	5/50	5/125	2/108	2/24 (1st trial); 2/40 (2nd trial)
GM cultivar (No. of plots)	YieldGard^®^ MON 810 ^1^ (5)	YieldGard^®^ MON 810 ^1^ (5)	YieldGard VT Rootworm/RR2™ MON 88017 ^1^ (5)	Roundup Ready™ 2 NK 603 ^1^ (54 ^2^)	YieldGard^®^ MON 810 ^1^ (4 in 1st trial; 10 in 2nd trial)
Near-isogenic cultivar (No. of plots)	Monumental (5)	Monumental (5)	DK 315 (5, 5 ^3^)	None	DKC 3871 (4, 4 in 1st field trial; 10 in 2nd field trial ^4^)
Other treatments (No. of plots)	None	None	(b) Cultivar Kipous (KWS SAAT AG) (5) (c) Cv. PR38N86 (DuPont Pioneer) (5)	None	None
References	[48]	[10,49]	[50]	[46]	None

^1^ MONSANTO Technology LLC; ^2^ Treatments: Herbicides: (a) Foramsulfuron; (b) Glyphosate: split application; and (c) Glyphosate + acetochlor, Tillage: (a) Conventional; (b) Reduced; and (c) Cover crops: *Hordeum vulgare*, *Phacelia tanacetifolia*, *Sinapis alba* or *Trifolium incarnatum*; ^3^ Treatments: (a) DK 315 alone; and (b) DK 315 + insecticide chlorpyrifos; ^4^ Treatments: 1st trial: (a) DKC 3871 + lambda-cyhalothrin (0.25 L/ha); and (b) DKC 3871 + bioinsecticide *Bacillus thuringiensis* ssp. *kurstaki* (1.5 L/ha), 2nd trial: DKC 3871 + lambda-cyhalothrin.

## Supplementary Materials

The following are available online at www.mdpi.com/2072-6651/9/4/121/s1, Table S1: The sample dates of deployment of pitfall traps in the locality western Slovakia in 2014 and 2015, Table S2: The abundance of carabids in the examined localities in 2002–2015, Table S3: Incidence and functional traits of carabids captured in localities South Bohemia 1, 2 and 3, Central Bohemia and western Slovakia in 2002–2015, Table S4: Data for analysis of explained variability in SaS model, Table S5: Data for analysis of explained variability in SaT model, Table S6: Data for analysis of explained variability in TaT model.
