# Supplementary material for: Use of Carabids for the Post-Market Environmental Monitoring of Genetically Modified Crops
Source: Toxins (Basel). 2017 Mar 29;9(4):121. doi: 10.3390/toxins9040121 (PMC5408195; doi:10.3390/toxins9040121)
Supplement: Supplementary file 1 [file toxins-09-00121-s001.zip › toxins-174315 Tables S1-S3.docx]

Supplementary Materials: Use of Carabids for the Post-Market Environmental Monitoring of Genetically Modified Crops

**Oxana Skoková Habuštová, Zdeňka Svobodová, Ľudovít Cagáň and František Sehnal**

**Table S1.** The sample dates of deployment of pitfall traps at the locality western Slovakia
in 2014 and 2015.

| **2014** | **2015** | **Stage of maize** |
| --- | --- | --- |
| May 15 | May 21 | BBCH 09 |
| June 10 | June 04 | BBCH 11 |
| June 24 | June 18 | BBCH 13 |
| July 8 | July 01 | BBCH 17 |
| July 22 | July 16 | BBCH 34 |
| August 5 | July 30 | BBCH 53 |
| August 19 | August 12 | BBCH 63 |
| September 3 | August 27 | BBCH 69 |
| September 16 | September 10 | BBCH 79 |
| October 1 | September 24 | BBCH 89 |

**Table S2.** The abundance of carabids in the examined localities in 2002–2015.

| **Tribe** | **Species** | **Localities** | | | | | **Total** |
| --- | --- | --- | --- | --- | --- | --- | --- |
|  |  | **South Bohemia 1** | **South Bohemia 2** | **South Bohemia 3** | **Central Bohemia** | **western Slovakia** |  |
| Pterostichini | *Abax parallelus* (Duftschmid, 1812) | 0 | 0 | 0 | 1 | 0 | 1 |
| Harpalini | *Acupalpus meridianus* (Linnaeus, 1761) | 0 | 0 | 0 | 1 | 1 | 2 |
| Platynini | *Agonum gracilipes* (Duftschmid, 1812) | 0 | 11 | 3 | 0 | 0 | 14 |
| Platynini | *Agonum muelleri* (Herbst, 1784*)* | 42 | 211 | 3 | 0 | 0 | 256 |
| Platinini | *Agonum sexpunctatum* (Linnaeus, 1758) | 1 | 5 | 3 | 0 | 0 | 9 |
| Platynini | *Agonum versutum* (Sturm, 1824) | 0 | 0 | 1 | 0 | 0 | 1 |
| Zabrini | *Amara aenea* (De Geer, 1774) | 0 | 75 | 6 | 1 | 1 | 83 |
| Zabrini | *Amara apricaria* (Paykull, 1790) | 0 | 6 | 0 | 0 | 1 | 7 |
| Zabrini | *Amara aulica* (Panzer, 1796) | 2 | 2 | 3 | 0 | 0 | 7 |
| Zabrini | *Amara consularis* (Duftschmid 1812) | 3 | 1 | 0 | 139 | 0 | 143 |
| Zabrini | *Amara cursitans* (Zimmermann, 1831) | 1 | 5 | 0 | 0 | 0 | 6 |
| Zabrini | *Amara eurynota* (Panzer, 1797) | 1 | 55 | 0 | 0 | 0 | 56 |
| Zabrini | *Amara familiaris* (Duftschmid 1812) | 0 | 8 | 0 | 0 | 0 | 8 |
| Zabrini | *Amara littorea (C.G. Thomson, 1857)* | 1 | 6 | 0 | 0 | 0 | 7 |
| Zabrini | *Amara nitida* (Sturm, 1825) | 0 | 1 | 0 | 0 | 0 | 1 |
| Zabrini | *Amara ovata* (Fabricius, 1792) | 3 | 8 | 0 | 6 | 0 | 17 |
| Zabrini | *Amara similata* (Gyllenhal, 1810) | 0 | 59 | 0 | 0 | 0 | 59 |
| Zabrini | *Amara tibialis (Paykull, 1798)* | 0 | 2 | 0 | 0 | 0 | 2 |
| Platynini | *Anchomenus dorsalis* (Pontoppidan, 1763) | 3 | 3 | 10 | 12 | 1071 | 1099 |
| Harpalini | *Anisodactylus binotatus* (Fabricius, 1787) | 0 | 0 | 2 | 0 | 3 | 5 |
| Harpalini | *Anisodactylus signatus* (Panzer, 1797) | 0 | 3 | 1 | 2 | 1 | 7 |
| Licinini | *Badister bullatus* (Schrank, 1798) | 0 | 0 | 1 | 3 | 0 | 4 |
| Licinini | *Badister lacertosus* (Sturm, 1815) | 0 | 2 | 0 | 0 | 0 | 2 |
| Licinini | *Badister unipustulatus* (Bonelli, 1813) | 0 | 1 | 0 | 0 | 0 | 1 |
| Bembidiini | *Bembidion biguttatum* (Fabricius, 1779) | 0 | 3 | 0 | 0 | 0 | 3 |
| Bembidiini | *Bembidion femoratum* (Sturm, 1825) | 20 | 1 | 2 | 0 | 0 | 23 |
| Bembidiini | *Bembidion guttula* (Fabricius, 1792) | 0 | 1 | 0 | 0 | 0 | 1 |
| Bembidiini | *Bembidion illigeri* (Netolitzky, 1914) | 0 | 2 | 0 | 0 | 0 | 2 |
| Bembidiini | *Bembidion lampros* (Herbst, 1784) | 218 | 163 | 18 | 63 | 0 | 462 |
| Bembidiini | *Bembidion obtusum* (Audinet-Serville, 1821) | 0 | 0 | 0 | 45 | 1 | 46 |
| Bembidiini | *Bembidion properans* (Stephens, 1828) | 34 | 34 | 2 | 41 | 3 | 114 |

**Table S2.** *Cont.*

| **Tribe** | **Species** | **Localities** | | | | | **Total** |
| --- | --- | --- | --- | --- | --- | --- | --- |
|  |  | **South Bohemia 1** | **South Bohemia 2** | **South Bohemia 3** | **Central Bohemia** | **western Slovakia** |  |
| Bembidiini | *Bembidion quadrimaculatum* (Linnaeus, 1761) | 813 | 728 | 80 | 59 | 0 | 1680 |
| Brachinini | *Brachinus crepitans* (Linnaeus, 1758) | 0 | 0 | 0 | 13 | 335 | 348 |
| Brachinini | *Brachinus ganglbaueri* (Schauberger, 1921) | 0 | 0 | 0 | 0 | 294 | 294 |
| Brachinini | *Brachinus explodens* (Duftschmid, 1812) | 0 | 0 | 0 | 0 | 71 | 71 |
| Brachinini | *Brachinus psophia* (Audinet-Serville 1821) | 0 | 0 | 0 | 0 | 31 | 31 |
| Platynini | *Calathus ambiguus* (Paykull, 1790) | 13 | 3 | 0 | 1 | 14 | 31 |
| Platynini | *Calathus erratus* (C. R. Sahlberg, 1827) | 0 | 6 | 0 | 2 | 0 | 8 |
| Platynini | *Calathus fuscipes* (Goeze, 1777) | 814 | 566 | 473 | 804 | 154 | 2811 |
| Platynini | *Calathus melanocephalus* (Linnaeus, 1758) | 71 | 7 | 13 | 0 | 0 | 91 |
| Carabini | *Calosoma auropunctatum* (Herbst, 1784) | 0 | 0 | 0 | 0 | 2 | 2 |
| Carabini | *Carabus granulatus* (Linnaeus, 1758) | 2 | 493 | 101 | 0 | 0 | 596 |
| Carabini | *Carabus hortensis* (Linnaeus, 1758) | 0 | 2 | 0 | 0 | 0 | 2 |
| Carabini | *Carabus scheidleri* (Panzer, 1799) | 36 | 1 | 0 | 0 | 0 | 37 |
| Carabini | *Carabus violaceus* (Linnaeus, 1758) | 0 | 11 | 2 | 0 | 1 | 14 |
| Callistini | *Chlaenius nitidulus* (Schrank, 1781) | 0 | 2 | 0 | 0 | 0 | 2 |
| Clivinini | *Clivina fossor* (Linnaeus, 1758) | 161 | 135 | 29 | 0 | 0 | 325 |
| Platynini | *Dolichus halensis* (Schaller, 1783) | 0 | 0 | 0 | 42 | 52 | 94 |
| Harpalini | *Harpalus affinis* (Schrank, 1781) | 97 | 484 | 117 | 60 | 162 | 920 |
| Harpalini | *Harpalus caspius roubali* (Schauberger, 1928) | 0 | 0 | 0 | 0 | 1 | 1 |
| Harpalini | *Harpalus distinguendus* (Duftschmid, 1812) | 0 | 20 | 0 | 124 | 60 | 204 |
| Harpalini | *Harpalus honestus* (Duftschmid, 1812) | 0 | 0 | 0 | 3 | 0 | 3 |
| Harpalini | *Harpalus rubripes* (Dufischmid, 1812) | 0 | 8 | 2 | 2724 | 0 | 2734 |
| Harpalini | *Harpalus signaticornis* (Duftschmid in 1812) | 0 | 1 | 0 | 1 | 0 | 2 |
| Harpalini | *Harpalus smaragdinus* (Duftschmid in 1812) | 1 | 1 | 0 | 0 | 0 | 2 |
| Harpalini | *Harpalus tardus* (Panzer, 1797) | 0 | 0 | 0 | 0 | 1 | 1 |
| Platynini | *Laemostenus terricola* (Herbst, 1784) | 0 | 0 | 0 | 0 | 38 | 38 |
| Trechini | *Lasiotrechus discus* (Fabricius, 1792) | 1 | 0 | 0 | 0 | 0 | 1 |
| Nebrini | *Leistus ferrugineus* (Linnaeus, 1758) | 0 | 0 | 0 | 0 | 15 | 15 |
| Loricerini | *Loricera pilicornis* (Fabricius, 1775) | 37 | 2 | 11 | 1 | 0 | 51 |
| Lebiini | *Microlestes maurus* (Sturm, 1827) | 4 | 16 | 1 | 12 | 4 | 37 |
| Lebiini | *Microlestes minutulus* (Goeze 1777) | 3 | 8 | 0 | 0 | 0 | 11 |

**Table S2.** *Cont.*

| **Tribe** | **Species** | **Localities** | | | | | **Total** |
| --- | --- | --- | --- | --- | --- | --- | --- |
|  |  | **South Bohemia 1** | **South Bohemia 2** | **South Bohemia 3** | **Central Bohemia** | **western Slovakia** |  |
| Nebtriini | *Nebria brevicollis* (Fabricius, 1792) | 19 | 0 | 1 | 0 | 0 | 20 |
| Notiophilini | *Notiophilus aquaticus* (Linnaeus, 1758) | 0 | 1 | 1 | 0 | 0 | 2 |
| Notiophilini | *Notiophilus palustris* (Duftschmid, 1812) | 3 | 0 | 0 | 59 | 1 | 63 |
| Notiophilini | *Notiophilus pusillus* (G.R.Waterhouse, 1833) | 0 | 0 | 0 | 13 | 0 | 13 |
| Harpalini | *Ophonus azureus* (Fabricius, 1775) | 0 | 2 | 1 | 0 | 0 | 3 |
| Harpalini | *Ophonus nitidulus* (Stephens, 1828) | 0 | 0 | 0 | 1 | 0 | 1 |
| Platynini | *Platynus assimilis* (Paykull, 1790) | 0 | 2 | 1 | 0 | 0 | 3 |
| Pterostichini | *Poecilus cupreus* (Linnaeus, 1758) | 874 | 13 234 | 1467 | 250 | 150 | 15 975 |
| Pterostichini | *Poecilus lepidus* (Leske 1785) | 0 | 0 | 0 | 0 | 63 | 63 |
| Pterostichini | *Poecilus sericeus* (Fischer von Waldheim, 1824) | 0 | 0 | 0 | 0 | 77 | 77 |
| Pterostichini | *Poecilus versicolor* (Sturm, 1824) | 1 | 1499 | 207 | 3 | 0 | 1710 |
| Pterostichini | *Pseudoophonus griseus* (Panzer, 1796) | 0 | 17 | 0 | 5 | 0 | 22 |
| Pterostichini | *Pseudoophonus rufipes* (De Geer) | 239 | 932 | 144 | 0 | 6556 | 7871 |
| Pterostichini | *Pterostichus melanarius* (Illiger, 1798) | 1740 | 2978 | 12 506 | 995 | 78 | 18 297 |
| Pterostichini | *Pterostichus melas* (Creutzer, 1799) | 0 | 0 | 0 | 0 | 1 | 1 |
| Pterostichini | *Pterostichus niger* (Schaller, 1783) | 2 | 87 | 21 | 0 | 1 | 111 |
| Pterostichini | *Pterostichus nigrita* (Paykull, 1790) | 0 | 52 | 11 | 0 | 0 | 63 |
| Pterostichini | *Pterostichus ovoideus* (Sturm, 1824) | 0 | 2 | 0 | 0 | 0 | 2 |
| Pterostichini | *Pterostichus strenuus* (Panzer, 1796) | 13 | 10 | 0 | 0 | 0 | 23 |
| Pterostichini | *Pterostichus vernalis* (Panzer, 1795) | 7 | 3 | 0 | 1 | 0 | 11 |
| Pterostichini | *Stomis pumicatus* (Panzer, 1795) | 0 | 0 | 0 | 4 | 0 | 4 |
| Trechini | *Trechus quadristriatus* (Schrank, 1781) | 204 | 33 | 329 | 160 | 115 | 841 |
| Harpalini | *Trichotichnus laevicollis* (Duftschmid, 1812) | 0 | 1 | 0 | 0 | 0 | 1 |
| Zabrini | *Zabrus tenebrioides* (Goeze, 1777) | 0 | 0 | 0 | 180 | 42 | 222 |

**Table S3.** Incidence and functional traits of carabids^1^ captured in localities South Bohemia 1, 2 and 3, Central Bohemia and western Slovakia in 2002–2015.

| **Tribe** | **Species** | **Incidence** | **Body size** | **Habitat affinity** | **Humidity affinity** | **Breeding period** | **Food specialization** |
| --- | --- | --- | --- | --- | --- | --- | --- |
| Pterostichini | *Abax parallelus* (Duftschmid, 1812) | Least concern | B | Silvicolous | Hygrophilous | Spring | Carnivorous |
| Harpalini | *Acupalpus meridianus* (Linnaeus, 1761) | Least concern | D | Open biotopes | Mesophilous | Spring | Omnivorous |
| Platynini | *Agonum gracilipes* (Duftschmid, 1812) | Least concern | C | Silvicolous | Eurytopic | Spring | Carnivorous |
| Platynini | *Agonum muelleri* (Herbst, 1784*)* | Least concern | C | Eurytopic | Hygrophilous | Spring | Carnivorous |
| Platinini | *Agonum sexpunctatum* (Linnaeus, 1758) | Least concern | C | Eurytopic | Hygrophilous | Spring | Carnivorous |
| Platynini | *Agonum versutum* (Sturm, 1824) | Least concern | C | Silvicolous | Hygrophilous | Spring | Carnivorous |
| Zabrini | *Amara aenea* (De Geer, 1774) | Least concern | C | Open biotopes | Xerophilous | Spring | Omnivorous |
| Zabrini | *Amara apricaria* (Paykull, 1790) | Least concern | C | Open biotopes | Xerophilous | Autumn | Omnivorous |
| Zabrini | *Amara aulica* (Panzer, 1796) | Least concern | B | Open biotopes | Mesophilous | Autumn | Omnivorous |
| Zabrini | *Amara consularis* (Duftschmid 1812) | Least concern | C | Eurytopic | Eurytopic | Autumn | Omnivorous |
| Zabrini | *Amara cursitans* (Zimmermann, 1831) | Least concern | C | Eurytopic | Eurytopic | Autumn | Omnivorous |
| Zabrini | *Amara eurynota* (Panzer, 1797) | Least concern | C | Open biotopes | Mesophilous | Spring | Omnivorous |
| Zabrini | *Amara familiaris* (Duftschmid 1812) | Least concern | C | Open biotopes | Mesophilous | Spring | Omnivorous |
| Zabrini | *Amara littorea* (*C.G. Thomson, 1857)* | Least concern | C | Open biotopes | Xerophilous | Spring | Omnivorous |
| Zabrini | *Amara nitida* (Sturm, 1825) | Least concern | C | Open biotopes | Xerophilous | Spring | Omnivorous |
| Zabrini | *Amara ovata* (Fabricius, 1792) | Least concern | C | Silvicolous | Hygrophilous | Spring | Omnivorous |

**Table S3.** *Cont.*

| **Tribe** | **Species** | **Incidence** | **Body size** | **Habitat affinity** | **Humidity affinity** | **Breeding period** | **Food specialization** |
| --- | --- | --- | --- | --- | --- | --- | --- |
| Zabrini | *Amara similata* (Gyllenhal, 1810) | Least concern | C | Open biotopes | Mesophilous | Spring | Omnivorous |
| Zabrini | *Amara tibialis (Paykull, 1798)* | Least concern | D | Open biotopes | Mesophilous | Spring/summer | Omnivorous |
| Platynini | *Anchomenus dorsalis* (Pontoppidan, 1763) | Least concern | C | Open biotopes | Hygrophilous | Spring | Carnivorous |
| Harpalini | *Anisodactylus binotatus* (Fabricius, 1787) | Least concern | C | Open biotopes | Mesophilous | Spring | Omnivorous |
| Harpalini | *Anisodactylus signatus* (Panzer, 1797) | Least concern | B | Open biotopes | Hygrophilous | Spring | Omnivorous |
| Licinini | *Badister bullatus* (Schrank, 1798) | Least concern | D | Eurytopic | Hygrophilous | Spring | Carnivorous |
| Licinini | *Badister lacertosus* (Sturm, 1815) | Least concern | C | Silvicolous | Hygrophilous | Spring | Carnivorous |
| Licinini | *Badister unipustulatus* (Bonelli, 1813) | Least concern | C | Silvicolous | Hygrophilous | Spring | Carnivorous |
| Bembidiini | *Bembidion biguttatum* (Fabricius, 1779) | Least concern | D | Silvicolous | Hygrophilous | Spring | Carnivorous |
| Bembidiini | *Bembidion femoratum* (Sturm, 1825) | Least concern | D | Eurytopic | Mesophilous | Spring | Carnivorous |
| Bembidiini | *Bembidion guttula* (Fabricius, 1792) | Least concern | D | Eurytopic | Hygrophilous | Spring | Carnivorous |
| Bembidiini | *Bembidion illigeri* (Netolitzky, 1914) | Least concern | D | Eurytopic | Hygrophilous | Spring | Carnivorous |
| Bembidiini | *Bembidion lampros* (Herbst, 1784) | Least concern | D | Open biotopes | Eurytopic | Spring | Carnivorous |
| Bembidiini | *Bembidion obtusum* (Audinet-Serville, 1821) | Least concern | D | Eurytopic | Eurytopic | Autumn/early spring | Carnivorous |
| Bembidiini | *Bembidion properans* (Stephens, 1828) | Least concern | D | Open biotopes | Hygrophilous | Spring | Carnivorous |
| Bembidiini | *Bembidion quadrimaculatum* (Linnaeus, 1761) | Least concern | D | Open biotopes | Eurytopic | Spring | Carnivorous |

**Table S3.** *Cont.*

| **Tribe** | **Species** | **Incidence** | **Body size** | **Habitat affinity** | **Humidity affinity** | **Breeding period** | **Food specialization** |
| --- | --- | --- | --- | --- | --- | --- | --- |
| Brachinini | *Brachinus crepitans* (Linnaeus, 1758) | Least concern | C | Open biotopes | Xerophilous | Summer | Carnivorous |
| Brachinini | *Brachinus ganglbaueri* (Schauberger, 1921) | Least concern | C | Open biotopes | Hygrophilous | Spring | Carnivorous |
| Brachinini | *Brachinus explodens* (Duftschmid, 1812) | Least concern | D | Open biotopes | Xerophilous | Spring | Carnivorous |
| Brachinini | *Brachinus psophia* (Audinet-Serville 1821) | CZ: endangered SK: least concern | C | Open biotopes | Hygrophilous | Spring | Carnivorous |
| Platynini | *Calathus ambiguus* (Paykull, 1790) | Least concern | C | Open biotopes | Xerophilous | Autumn | Carnivorous |
| Platynini | *Calathus erratus* (C. R. Sahlberg, 1827) | Least concern | C | Open biotopes | Xerophilous | Autumn | Carnivorous |
| Platynini | *Calathus fuscipes* (Goeze, 1777) | Least concern | B | Open biotopes | Xerophilous | Autumn | Carnivorous |
| Platynini | *Calathus melanocephalus* (Linnaeus, 1758) | Least concern | C | Open biotopes | Mesophilous | Autumn | Carnivorous |
| Carabini | *Calosoma auropunctatum* (Herbst, 1784) | CZ: vulnerable SK: endangered | A | Open biotopes | Xerophilous | Spring | Carnivorous |
| Carabini | *Carabus granulatus* (Linnaeus, 1758) | Least concern | B | Silvicolous | Hygrophilous | Spring | Carnivorous |
| Carabini | *Carabus hortensis* (Linnaeus, 1758) | Least concern | A | Silvicolous | Hygrophilous | Autumn | Carnivorous |
| Carabini | *Carabus scheidleri* (Panzer, 1799) | Least concern | A | Silvicolous | Eurytopic | Autumn | Carnivorous |
| Carabini | *Carabus violaceus* (Linnaeus, 1758) | Least concern | A | Silvicolous | Eurytopic | Autumn | Carnivorous |
| Callistini | *Chlaenius nitidulus* (Schrank, 1781) | Least concern | B | Open biotopes | Hygrophilous | Spring | Carnivorous |
| Clivinini | *Clivina fossor* (Linnaeus, 1758) | Least concern | C | Open biotopes | Hygrophilous | Spring | Carnivorous |
| Platynini | *Dolichus halensis* (Schaller, 1783) | Least concern | B | Open biotopes | Xerophilous | Late summer/ autumn | Carnivorous |

**Table S3.** *Cont.*

| **Tribe** | **Species** | **Incidence** | **Body size** | **Habitat affinity** | **Humidity affinity** | **Breeding period** | **Food specialization** |
| --- | --- | --- | --- | --- | --- | --- | --- |
| Harpalini | *Harpalus affinis*  (Schrank, 1781) | Least concern | C | Open biotopes | Eurytopic | Spring/summer/ autumn | Omnivorous |
| Harpalini | *Harpalus caspius roubali* (Schauberger, 1928) | Least concern | B | Open biotopes | Xerophilous | Autumn | Omnivorous |
| Harpalini | *Harpalus distinguendus* (Duftschmid, 1812) | Least concern | C | Open biotopes | Eurytopic | Spring/summer | Omnivorous |
| Harpalini | *Harpalus honestus* (Duftschmid, 1812) | Least concern | C | Open biotopes | Xerophilous | Spring | Omnivorous |
| Harpalini | *Harpalus rubripes* (Dufischmid, 1812) | Least concern | C | Open biotopes | Eurytopic | Spring | Omnivorous |
| Harpalini | *Harpalus signaticornis* (Duftschmid in 1812) | Least concern | C | Open biotopes | Xerophilous | Spring/autumn | Omnivorous |
| Harpalini | *Harpalus smaragdinus* (Duftschmid in 1812) | Least concern | C | Open biotopes | Xerophilous | Spring/autumn | Omnivorous |
| Harpalini | *Harpalus tardus* (Panzer, 1797) | Least concern | C | Open biotopes | Eurytopic | Spring | Omnivorous |
| Platynini | *Laemostenus terricola* (Herbst, 1784) | CZ: near threatened SK: least concern | B | Open biotopes | Xerophilous | Autumn | Carnivorous |
| Trechini | *Lasiotrechus discus* (Fabricius, 1792) | Least concern | D | Eurytopic | Hygrophilous | Spring | Carnivorous |
| Nebrini | *Leistus ferrugineus* (Linnaeus, 1758) | Least concern | C | Eurytopic | Hygrophilous | Autumn | Carnivorous |
| Loricerini | *Loricera pilicornis* (Fabricius, 1775) | Least concern | C | Eurytopic | Hygrophilous | Spring/summer | Carnivorous |
| Lebiini | *Microlestes maurus* (Sturm, 1827) | Least concern | D | Eurytopic | Eurytopic | Spring | Omnivorous |
| Lebiini | *Microlestes minutulus* (Goeze 1777) | Least concern | D | Eurytopic | Eurytopic | Spring | Carnivorous |
| Nebtriini | *Nebria brevicollis* (Fabricius, 1792) | Least concern | B | Eurytopic | Hygrophilous | Autumn | Carnivorous |
| Notiophilini | *Notiophilus aquaticus* (Linnaeus, 1758) | Least concern | D | Silvicolous | Hygrophilous | Spring/summer | Carnivorous |

**Table S3.** *Cont.*

| **Tribe** | **Species** | **Incidence** | **Body size** | **Habitat affinity** | **Humidity affinity** | **Breeding period** | **Food specialization** |
| --- | --- | --- | --- | --- | --- | --- | --- |
| Notiophilini | *Notiophilus palustris* (Duftschmid, 1812) | Least concern | D | Silvicolous | Hygrophilous | Spring | Carnivorous |
| Notiophilini | *Notiophilus pusillus* (G.R.Waterhouse, 1833) | Least concern | D | Eurytopic | Eurytopic | Spring | Carnivorous |
| Harpalini | *Ophonus azureus* (Fabricius, 1775) | Least concern | C | Open biotopes | Xerophilous | Autumn | Granivorous |
| Harpalini | *Ophonus nitidulus* (Stephens, 1828) | Least concern | C | Open biotopes | Xerophilous | Spring | Granivorous |
| Platynini | *Platynus assimilis* (Paykull, 1790) | Least concern | C | Silvicolous | Hygrophilous | Spring | Carnivorous |
| Pterostichini | *Poecilus cupreus* (Linnaeus, 1758) | Least concern | B | Eurytopic | Eurytopic | Spring | Omnivorous |
| Pterostichini | *Poecilus lepidus* (Leske 1785) | Least concern | B | Eurytopic | Xerophilous | Spring | Carnivorous |
| Pterostichini | *Poecilus sericeus* (Fischer von Waldheim, 1824) | CZ: vulnerable SK: least concern | B | Eurytopic | Mesophilous | Spring | Carnivorous |
| Pterostichini | *Poecilus versicolor* (Sturm, 1824) | Least concern | C | Open biotopes | Hygrophilous | Spring | Carnivorous |
| Pterostichini | *Pseudoophonus griseus* (Panzer, 1796) | Least concern | C | Open biotopes | Mesophilous | Autumn | Omnivorous |
| Pterostichini | *Pseudoophonus rufipes* (De Geer) | Least concern | B | Open biotopes | Eurytopic | Autumn | Omnivorous |
| Pterostichini | *Pterostichus melanarius* (Illiger, 1798) | Least concern | B | Eurytopic | Mesophilous | Autumn | Carnivorous |
| Pterostichini | *Pterostichus melas* (Creutzer, 1799) | Least concern | B | Silvicolous | Mesophilous | Autumn | Carnivorous |
| Pterostichini | *Pterostichus niger* (Schaller, 1783) | Least concern | B | Silvicolous | Hygrophilous | Autumn | Carnivorous |
| Pterostichini | *Pterostichus nigrita* (Paykull, 1790) | Least concern | B | Eurytopic | Hygrophilous | Spring | Carnivorous |
| Pterostichini | *Pterostichus ovoideus* (Sturm, 1824) | Least concern | C | Silvicolous | Hygrophilous | Spring | Carnivorous |

**Table S3.** *Cont.*

| **Tribe** | **Species** | **Incidence** | **Body size** | **Habitat affinity** | **Humidity affinity** | **Breeding period** | **Food specialization** |
| --- | --- | --- | --- | --- | --- | --- | --- |
| Pterostichini | *Pterostichus strenuus* (Panzer, 1796) | Least concern | C | Silvicolous | Hygrophilous | Spring | Carnivorous |
| Pterostichini | *Pterostichus vernalis* (Panzer, 1795) | Least concern | C | Eurytopic | Hygrophilous | Spring/summer | Carnivorous |
| Pterostichini | *Stomis pumicatus* (Panzer, 1795) | Least concern | C | Eurytopic | Hygrophilous | Spring | Carnivorous |
| Trechini | *Trechus quadristriatus* (Schrank, 1781) | Least concern | D | Open biotopes | Mesophilous | Autumn | Carnivorous |
| Harpalini | *Trichotichnus laevicollis* (Duftschmid, 1812) | Least concern | C | Silvicolous | Mesophilous | Spring/autumn | Omnivorous |
| Zabrini | *Zabrus tenebrioides* (Goeze, 1777) | Least concern | B | Open biotopes | Xerophilous | Autumn | Omnivorous |

^1^ The tribe, species, body size, habitat and humidity affinity, breeding period [1], incidence [2,3] and food specializations [4] were determined. Body size (mid-range): A: ˃22 mm, B: 11–21.9 mm, C: 6–10.9 mm, D: ˂5.9 mm; Silvicolous: preferring woodlands; Open biotopes: preferring open areas; Eurytopic: adaptable to various environmental conditions; Hygrophilous: preferring moist places; Mesophilous: preferring intermediate or moderate environmental conditions, avoid extremes of moisture or dryness; Xerophilous: preferring dry environmental conditions.

References

1. Hůrka, K. *Carabidae of the Czech and Slovak Republics*; Kabourek: Zlín, Czech Republic, 1996; p. 565.
2. Veselý, P.; Moravec, P.; Stanovský, J. Carabidae (střevlíkovití). In *Červený Seznam Ohrožených Druhů České Republiky*; Farkáč, J., Král, D., Škorpík, M., Eds.; AOPK: Praha, Czech Republic, 2005; pp. 406–411.
3. Holecová, M.; Franc, V. Red (Ecosozological) list of beetles (Coleoptera) of Slovakia. In *Red List of Plants and Animals of Slovakia*. *Ochr. Prír.* **2001**, *20* (Supp.), 111–128.
4. Larochelle, A. The food of carabid beetles (Coleoptera: Carabidae, including Cicindelinae). *Fabreries* **1990**, *5*, 1–132.
